# Hydrogen peroxide – production, fate and role in redox signaling of tumor cells

**DOI:** 10.1186/s12964-015-0118-6

**Published:** 2015-09-14

**Authors:** Claudia Lennicke, Jette Rahn, Rudolf Lichtenfels, Ludger A. Wessjohann, Barbara Seliger

**Affiliations:** Institute of Medical Immunology, Martin Luther University Halle-Wittenberg, Magdeburger Str. 2, 06112 Halle/Saale, Germany; Leibniz-Institute of Plant Biochemistry, Weinberg 3, 06120 Halle /Saale, Germany

## Abstract

Hydrogen peroxide (H_2_O_2_) is involved in various signal transduction pathways and cell fate decisions. The mechanism of the so called “redox signaling” includes the H_2_O_2_-mediated reversible oxidation of redox sensitive cysteine residues in enzymes and transcription factors thereby altering their activities. Depending on its intracellular concentration and localization, H_2_O_2_ exhibits either pro- or anti-apoptotic activities. In comparison to normal cells, cancer cells are characterized by an increased H_2_O_2_ production rate and an impaired redox balance thereby affecting the microenvironment as well as the anti-tumoral immune response. This article reviews the current knowledge about the intracellular production of H_2_O_2_ along with redox signaling pathways mediating either the growth or apoptosis of tumor cells. In addition it will be discussed how the targeting of H_2_O_2_-linked sources and/or signaling components involved in tumor progression and survival might lead to novel therapeutic targets.

## Introduction

Hydrogen peroxide (H_2_O_2_) is next to the superoxide anion and hydroxyl radical a key member of the class of reactive oxygen species (ROS), which are in particular generated via the respiratory chain cascade but also as byproducts of the cellular metabolism including protein folding. In contrast to the superoxide anion and hydroxyl radical, the less reactive H_2_O_2_ is involved in many physiological processes such as hypoxic signal transduction, cell differentiation and proliferation but also plays a role in mediating immune responses. However, it exerts its effects depending on the cellular context, its local concentration as well as its exposure time [1, 2]. Thus H_2_O_2_ is no more considered as an unwanted rather toxic byproduct, but plays an important role in the control of vital cellular processes.

Tumor cells are characterized by an enhanced metabolic activity resulting in changes of the cellular redox state that has to handle the production of high levels of ROS [3]. In many cancer cells persistently upregulated H_2_O_2_-dependent signaling pathways are involved in cell differentiation, growth and survival, yet high levels of H_2_O_2_ can also induce cell cycle arrest or apoptosis in cells. Due to this dual functionality of H_2_O_2_ robust cellular anti-oxidative systems are thought to be essential for maintaining the cellular redox homeostasis. Several defense systems against oxidative stress have been shown to be upregulated in cancer cells via the transcription factor nuclear factor-erythroid 2 p45-related factor 2 (Nrf2) [4]. These include the thioredoxin/thioredoxin reductase (Trx/TrxR) system, peroxiredoxins (Prxs) and several glutathione S-transferases (GSTs), which are involved in mediating the cellular redox homeostasis, but still allow redox modifications of specific redox-sensitive proteins thereby triggering redox signaling events. In this review we will address how (i) cellular H_2_O_2_ is produced and how it regulates certain signaling pathways, (ii) tumor cells cope with enhanced H_2_O_2_ levels to escape from oxidative stress, (iii) potential redox-sensors might be correlated with tumorigenesis, and how (iv) H_2_O_2_-modulated processes/pathways might be used as therapeutic targets.

## Sources of H_2_O_2_

**Fig. 1:**
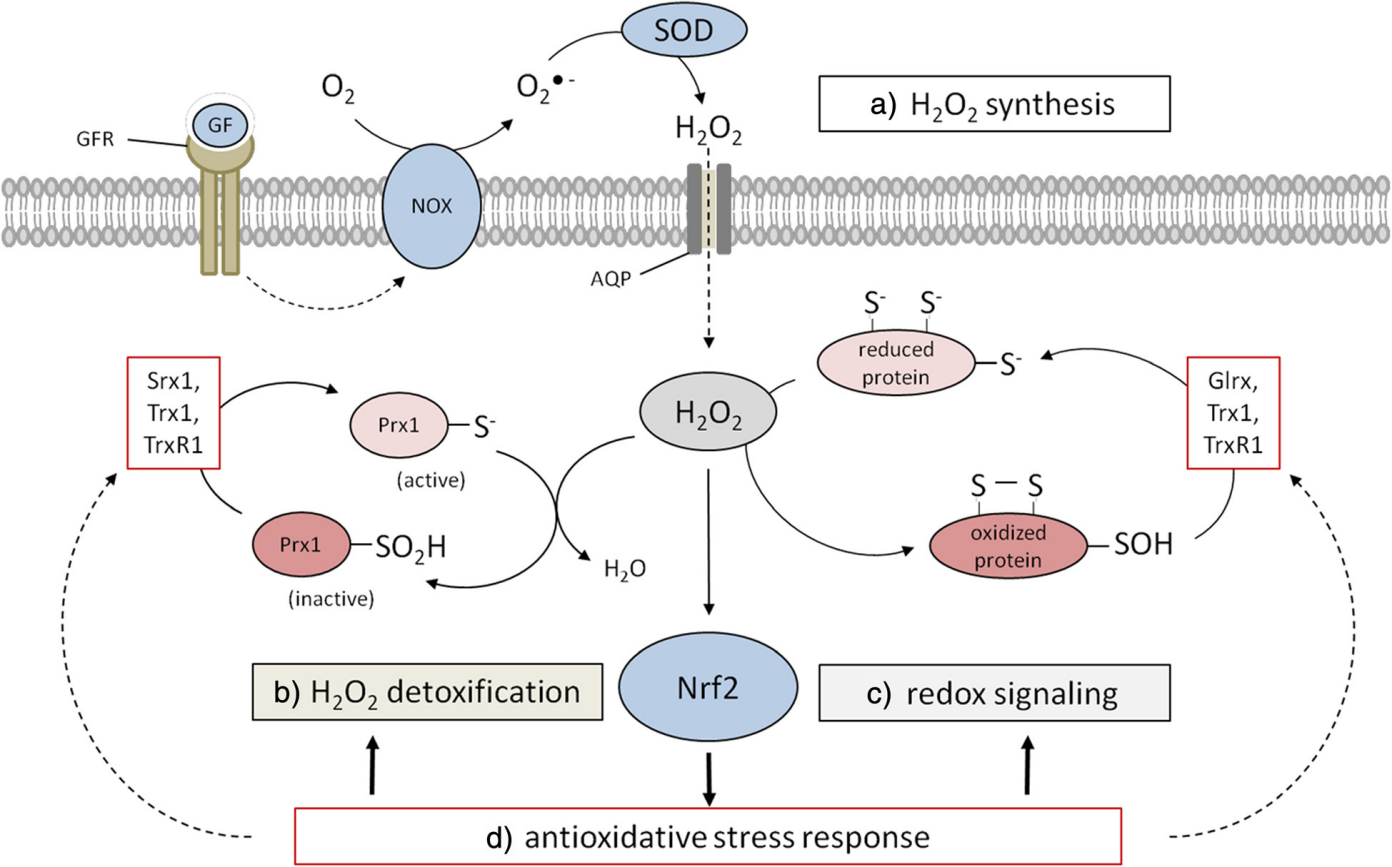
Interplay between physiological/pathophysiological H_2_O_2_ generation and the anti-oxidative response mechanism. **a** H_2_O_2_ is produced, e.g. in response to growth factors by the NOX/SOD system and enters cells through simple diffusion and facilitated diffusion through AQPs, respectively, leading to increased intracellular H_2_O_2_ levels. **b** Peroxiredoxins (Prx) act as highly active redox sensors and are part of one of the main H_2_O_2_ detoxifying systems. Hyperoxidation inactivates Prxs allowing **c** the oxidation of sensitive cysteine residues in cellular proteins including transcription factors. **d** The Nrf2 system is activated in response to increased H_2_O_2_ levels leading to the anti-oxidative response. AQP, aquaporin; GF, growth factor; GFR, growth factor receptor.

Reactive oxygen species (ROS) represent a class of oxygen-containing chemical compounds that are defined by their reactivity towards biological targets, including lipids, proteins and DNA [3]. The most prominent member of this class is the superoxide anion (O_2_^−^), largely produced by either the mitochondrial electron transport chain, in particular its complexes I, II and III, or by NAD(P)H oxidases (NOXs). The O_2_^−^ is rapidly converted to H_2_O_2_ by distinct superoxide dismutases (SODs) (Fig. 1) or to hydroxyl radicals (OH^●^) [5]. While O_2_^−^ released into the mitochondrial matrix is directly converted by SOD2 into the less reactive H_2_O_2_, O_2_^−^ released by the complex III into the mitochondrial intermembrane space can be exported via voltage-dependent anion channels (VDAC) into the cytosol followed by a SOD1-mediated conversion into H_2_O_2_ [6, 7]. In addition, cellular membrane-associated NOXs transferring electrons from NAD(P)H across cell membranes to molecular oxygen (O_2_) are producers of superoxide anions. Via NOX2 O_2_^−^ can be transported into the extracellular space, where it can be either converted to H_2_O_2_ by SOD3 or re-imported via chloride channels [8]. Given that SODs are characterized as highly efficient enzymes the intracellular (cytosol - SOD1, mitochondria - SOD2) and extracellular (SOD3) balance is shifted towards the formation of H_2_O_2_, which diffuses relatively free or is receptor-mediated transported across biological membranes [9] thereby acting as an intra- and intercellular signaling molecule (Fig. 1). In contrast, the hydroxyl radical (OH^●^) is considered as the most reactive ROS species. Due to its high reactivity towards lipids, proteins and DNA, it has a short half-life thereby limiting its diffusion but causing damage largely at its site of production.

## Transport and subcellular localization of hydrogen peroxide

In comparison to water, H_2_O_2_ possesses a reduced membrane permeability, which is influenced by the phosphorylation and glycosylation states of membrane proteins, the lipid composition (lipid rafts) and osmotic stretching of lipid bilayers [10–16]. Aquaporin (AQP) 8, but not the classical AQP1 facilitates the transport of H_2_O_2_ across membranes [17, 18]. Treatment of AQP3-overexpressing HeLa cells with H_2_O_2_ resulted in an enhanced phosphorylation of protein kinase B (AKT) [19], while overexpression of AQP8 increased intracellular H_2_O_2_ levels in leukemia cells in the presence of H_2_O_2_. Moreover, vascular endothelial growth factor (VEGF) signaling results in increased intracellular H_2_O_2_ levels, which can be reduced by silencing AQP8 [20]. Furthermore, silencing of AQP8 can inhibit the epidermal growth factor (EGF) mediated stimulation of tyrosine kinases. [21]. Thus, AQPs not only play important roles in the diffusion of H_2_O_2_ across membranes, but also on downstream signaling cascades. Furthermore, H_2_O_2_ detoxifying enzymes, such as glutathione peroxidases (GPxs), catalases and Prxs, can lead to rapidly decreasing intracellular H_2_O_2_ concentrations [9] thereby establishing the formation of H_2_O_2_ gradients resulting in selective and localized H_2_O_2_ signaling events. The inactivation of scavenger enzymes by H_2_O_2_ represents a mechanism that allows the selective enrichment (“flooding”) of a cellular area by H_2_O_2_ thereby promoting the H_2_O_2_-mediated oxidation of specific thiols within target proteins at this site [22, 23].

## Features of H_2_O_2_ – second messenger like characteristics and principles of redox modifications

**Fig. 2:**
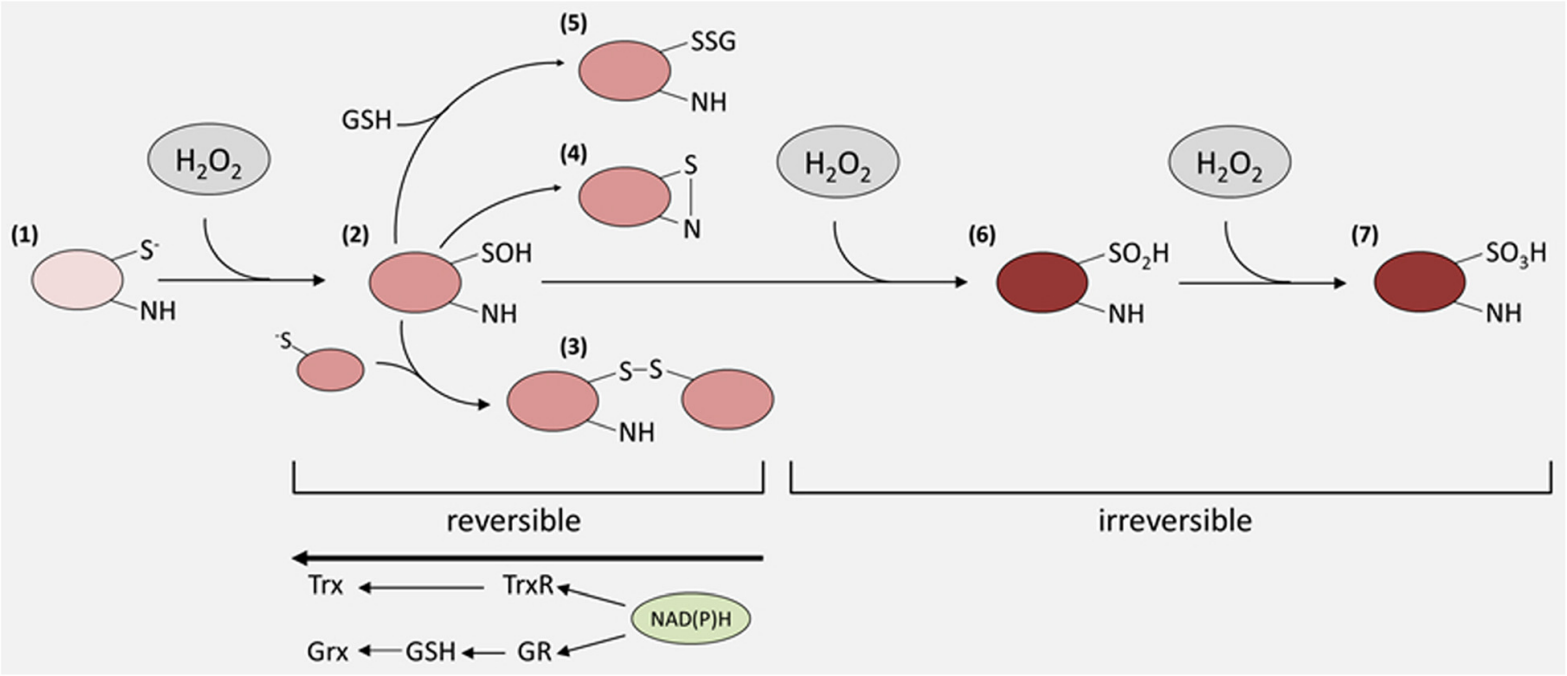
Redox modifications of reactive cysteine residues by H_2_O_2_. Redox-sensitive proteins contain cysteine residues, which are partially ionized under physiological pH. Oxidation of this thiolate anion (**1**) results in a sulfenic acid or rather its salt (**2**), which is relatively reactive and forms intra-/intermolecular disulfide bonds in the presence of thiolate. This sulfenylation can be intramolecular or intermolecular (**3**), the latter predominantly with GSH to form glutathionylated intermediates (**5**), or sulfenylamides with oxidizable amines (**4**) and glutathionylated intermediates (**5**), respectively. These redox modifications result in altered functions of their target proteins and can be reversed by the Trx- or GSH-based anti-oxidative systems. Under excessive H_2_O_2_ concentration the sulfonate or sulfonamide intermediates can be further irreversibly oxidized to sulfinic (**6**) and sulfonic acids (**7**) forming the respective anions under physiological pH thus also shifting the isoelectric points of affected proteins.

Since H_2_O_2_ is produced, enzymatically removed and exerts a low overall reactivity, but a relatively high selectivity towards certain proteins, in particular proteins containing thiol groups [24, 25], it is postulated to act as a second messenger. H_2_O_2_ mediates chemical modifications of specific cysteine residues, which are overrepresented in functionally relevant regions of some proteins [26]. Approximately 10 % of free cysteines are ionized at pH 7.4 due to their low pKa and thus are more susceptible to H_2_O_2_ than protonated cysteine thiol groups [27]. Although H_2_O_2_ detoxification enzymes, like GPxs, Prxs and catalase, are more abundantly expressed than proteins involved in the redox signaling [28], cysteine residues of the ubiquitously expressed Prxs are prone to be oxidized at even relatively low H_2_O_2_ levels [29–31]. In contrast, signaling molecules e.g. protein tyrosine phosphatases (PTPs) require extremely high concentrations of H_2_O_2_ to undergo oxidation [9]. Furthermore, if Prxs are inactivated by over-oxidation or phosphorylation this might lead to localized H_2_O_2_ accumulation thereby triggering redox signaling [22] (Fig. 1). The first step of oxidative thiol/thiolate modification by H_2_O_2_ is the formation of sulfonate or sulfenic acid (R-SOH), which might reacts with any thiol in the vicinity, e.g. glutathione (GSH) to form inter- and intramolecular disulfide bonds or protein-SSGs, respectively. In some cases, e.g. with electron-rich amino groups they also form sulfenylamides (Fig. 2). These oxidized forms can be easily reduced to thiolate by the Trx- and GSH-based anti-oxidative systems thereby ensuring the reversibility of redox modifications caused by H_2_O_2_. In the presence of excessive concentrations of H_2_O_2_ further oxidation of sulfenic acids might occur thereby resulting in the formation of sulfinic (−SO_2_H), sulfonic acids (−SO_3_H) or their respective anions. In general these sulfur (IV) and (VI) oxidative states are irreversible oxidation products and some of these species can be linked to H_2_O_2_-mediated toxicity (Fig. 2). However, if these oxidative modifications occur in members of the Prx family their reduction can be mediated by sulfiredoxins (Srx) [32], which might represent an adaptive process of eukaryotic cells to cope with increased H_2_O_2_ levels [22, 23].

## The anti-oxidative response – factors that maintain redox signaling

Whereas intracellular O_2_^−^ concentrations are tightly controlled by the activity of SODs and thus kept at very low levels [33], the metabolite H_2_O_2_ is a rather stable ROS compound. Cells have developed distinct mechanisms to maintain the production and clearance of such reactive species in a homeostatic state in order to properly proliferate and to differentiate. Tumor cells are metabolically hyperactive resulting in the production of excessive ROS levels including H_2_O_2_. To cope with enhanced H_2_O_2_ concentrations and to protect cells from oxidative damage, anti-oxidative defense systems are upregulated, which results in a shift of the redox balance towards an upregulation of pro-survival signaling pathways as summarized in Table 1 for a set of Nrf2-regulated anti-oxidative proteins and their correlation to cancer.

**Table 1:** Nrf2 targets and their correlation to cancer

target	name	relation to cancer
Trx1	thioredoxin 1	proto-oncogene expressed in many cancers [224]
		elevated serum levels in patients with ovarian cancer [225]
		overexpression in HCC, CRC, liver metastasis [224] and
		in breast cancer [226]
TrxR1	thioredoxin reductase1	overexpressed in many cancer cells and cell lines of distinct histology [227]
TXNIP	thioredoxin interacting protein	negatively interferes with bladder carcinogenesis [228]
		tumor suppressor in thyroid cancer [229]
Prx1	peroxiredoxin 1	increased expression in pancreatic cancer [230], breast cancer [226, 231] and HCC [232]
		promotion of lung cancer progression [233]
		related to tumor angiogenesis in pancreatic cancer [230]
Prx6	peroxiredoxin 6	promotion of lung tumor growth [234, 235] repressed expression in papillary thyroid carcinomas [236]
Srx	sulfiredoxin	oncogenic role in skin tumorigenesis [237]
		overexpression in squamous cell carcinoma [56]
		promotion of lung cancer progression [69]

*CRC* colorectal carcinoma, *HCC* hepatic cell carcinoma

### Transcription factor Nrf2 as regulator of the anti-oxidative response

**Fig. 3:**
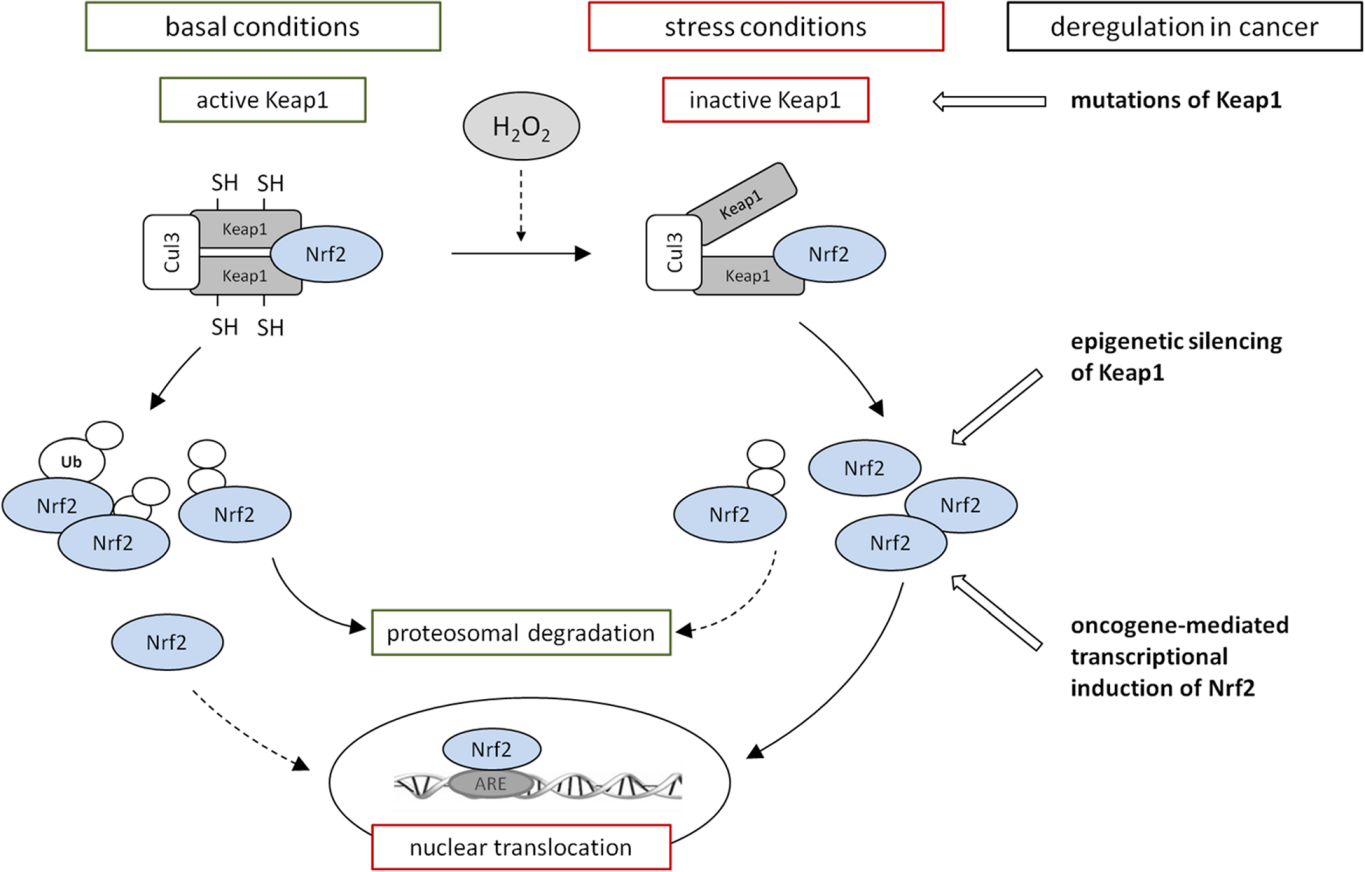
The Nrf2/Keap1 signaling pathway. Under basal conditions Nrf2 is bound by two molecules of Keap1, poly-ubiquitinylated by the Cul3 system and thereby marked for proteasomal degradation. Only a small portion of Nrf2 escapes from this degradation process and translocates to the nucleus to maintain the basal expression of anti-oxidant response genes. Under stress conditions like elevated levels of H_2_O_2_ Keap1 is modified at redox sensitive cysteine residues leading to an impaired conformation and inactivation of Keap1. Newly translated Nrf2 escapes ubiquitinylation, translocates to the nucleus and induces the anti-oxidative stress response. Mechanisms for the continuously accumulation of Nrf2 in the nucleus of several cancer cells can be triggered by (i) mutations of Keap1 associated with its inactivation, (ii) epigenetic silencing of Keap1 and (iii) mutations of oncogenes such as K-ras, B-raf and c-myc leading to the transcriptional induction of Nrf2.

**Fig. 4:**
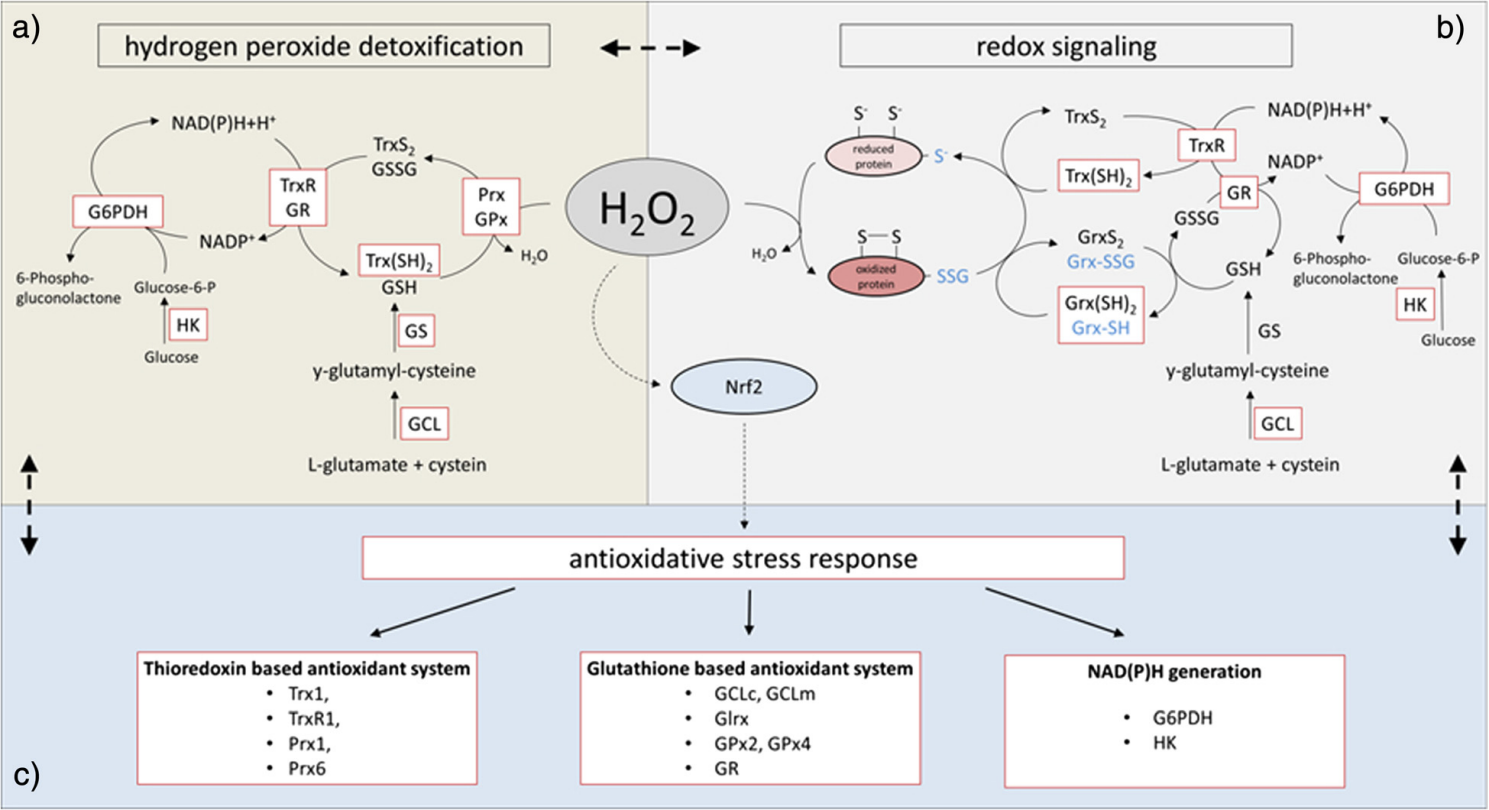
Maintenance of redox homeostasis by Nrf2. Nrf2 induces the expression of genes coding for enzymes involved in (**a**) hydrogen peroxide detoxification and (**b**) redox signaling. (**c**) High levels of H_2_O_2_ activate Nrf2 resulting in the induction of the anti-oxidative stress response. The red boxes symbolize Nrf2 inducible enzymes

Nuclear factor-erythroid 2 p45-related factor 2 (Nrf2) is a transcription factor (TF) that plays a key role in controlling the response to oxidative stress by its regulation of anti-oxidative enzymes, phase II enzymes and enzymes of the glutathione biosynthesis. Under physiological conditions the constitutive abundance of active Nrf2 is relatively low due to its continuous proteasomal degradation, but can be modified at the post-translational level to ensure rapid and efficient adaption to metabolic alterations in particular to oxidative stress. The best characterized repressor of Nrf2 is the kelch-like ECH-associated protein (Keap1), which serves as a substrate adapter protein within the RBX1 E3 ubiquitin ligase complex (CRL^Keap1^) [34]. Keap1 contains multiple highly reactive cysteine residues, which can act as stress sensors, if modified by electrophiles or oxidants, e.g. from food [35]. This results in an altered conformation of Keap1 and an impaired binding capacity to Nrf2 thereby preventing Nrf2 from proteasomal degradation. Thus, Nrf2 accumulates in the nucleus leading to the induction of genes by binding to the anti-oxidant response element (ARE) in their promoter regions (Fig. 3). In addition Nrf2 undergoes post-translational modifications such as PKC-dependent phosphorylation on Ser-40, phosphorylation through the MAPK/ERK signaling pathways in response to endoplasmic reticulum/unfolded protein stress or by casein kinase and CBP/p300 also promoting its binding to such ARE sites. The activation of Nrf2 can be also mediated by additional signal transduction pathways, e.g. ERK, c-Jun amino-terminal kinase (JNK), AMP-activated protein kinase (AMPK) or PI3K/AKT promoting anti-oxidative effects, which mediate enhanced resistance to oxidative stress as well as to further oxidative insults [36–38]. Constitutive stabilization of Nrf2 is found in several human cancers [39–41] and is associated with increased cancer chemotherapy resistance, enhanced tumor progression [42, 43] and poor prognosis and/or survival for patients [40, 44, 45]. Mechanisms by which the Nrf2 signaling pathway is constitutively activated in several types of cancer include (i) somatic mutations of Keap1 disrupting the binding capacity to Nrf2, (ii) epigenetic silencing of Keap1 and (iii) transcriptional induction of Nrf2 by oncogenes such as K-ras, B-raf or c-myc [46] (Fig. 3). Furthermore, increased levels of ROS (H_2_O_2_) and increased Nrf2 activity in tumor cells, result in an enhanced anaerobic glycolysis and utilization of the pentose phosphate pathway activity to generate NAD(P)H equivalents necessary for the Trx- and GSH-based anti-oxidative systems [47]. Since NAD(P)H generating enzymes are Nrf2 targets, the energy metabolism is directly connected with the redox homeostasis (Fig. 4). This is confirmed by an increased metabolic oxidative stress and cytotoxicity in response to the inhibition of glycolysis and/or the pentose phosphate pathways in combination with an inhibition of the Trx metabolism [48]. In contrast, knock down of Nrf2 suppresses tumor growth, inhibits cell proliferation and promotes increased apoptosis [44, 49]. The fact, that several cancers exhibit induced Nrf2 levels associated with enhanced tumor progression and chemotherapy resistance, whereas the lack of Nrf2 has opposite effects, Nrf2 represents a promising target for cancer therapies.

### Targets of Nrf2

#### Peroxiredoxins

Prxs represent members of the so called thiol-based anti-oxidant system [50] that act as redox switches to modulate homeostasis [51]. As important H_2_O_2_ scavenging enzymes Prxs are involved in the anti-oxidative response and in the regulation of redox-dependent signaling pathways by converting H_2_O_2_ into water [52, 53]. In mammals, the family of Prxs consists of 6 members located either in the cytosol (Prx1, Prx2, Prx4, Prx5, Prx6), mitochondria (Prx3, Prx5) or in other cellular compartments (Prx1, nucleus; Prx2, membrane; Prx4, Golgi apparatus, extracellular space, endoplasmic reticulum; Prx5, peroxisomes) [9, 54]. Prxs are upregulated under conditions of oxidative stress [55–57] and it could be shown that Prx1 and Prx6 are direct targets of Nrf2 [58, 59]. Prx1 – Prx5 are 2-Cys-Prx and utilize Trx as electron donor for their catalytic activity, while Prx6 is a 1-Cys-Prx and depends on GSH instead of Trx for its reduction [54, 60]. The hyper-oxidation of 2-Cys Prx, in particular of Prx1, adds further chaperone function to these Prxs, but depends on certain motif elements downstream of the peroxidatic cysteine residue (GGLG and YF motifs) [23, 61]. The chaperone function is based on the formation of stack like higher molecular weight complexes, thereby preventing the denaturation of proteins from external stresses like heat shock or oxidative stress. This multimeric complex can be subsequently dissolved into low molecular weight species by Srx [61]. Whereas in some species more distant cysteine residues might act as redox sensors, human Prxs are known to gain such a chaperone function only after the peroxidatic cysteine is hyper-oxidized [51]. At the transcriptional level Nrf2 and to some degree also focal adhesion kinase (FAK) have been demonstrated to activate the expression of Prxs [62, 63]. However, there is also evidence that modifications at the post-translational level have an impact on the function of Prxs. For example nitrosylation of the tyrosine residue within the YF motif of Prx2 plays a crucial role in the regulation of disulfide bond formation under oxidative stress conditions resulting in a more active and robust peroxidase [64]. In addition, its glutathionylation may affect its localization to the extracellular compartment, along with Trx, thereby inducing TNFα production leading to an oxidative stress-dependent inflammatory reaction [65]. For Prx3 the complex formation of FoxO3a with the peroxisome proliferator-activated receptor-gamma coactivator 1 alpha (PGC1 alpha) is enhanced by sirtuin-1 (SirT1), which is similar to the regulation of other anti-oxidant proteins [66]. The Prx4, which is mainly expressed in the endoplasmic reticulum compartment can be enhanced at the post-transcriptional level by calpain [67]. Due to its high susceptibility to undergo hyperoxidation even at low levels of oxidative stress its chaperone function is frequently involved in the oxidative folding of various ER resident proteins, likely in cooperation with protein disulfide isomerase (PDI) [68]. There is also evidence that Prx4 in addition to Srx plays a crucial role in enhancing RAS-RAF-MEK signaling to control cancer cell proliferation and metastasis formation [69].

#### Sulfiredoxins

Srxs reduce double oxidized catalytic cysteine (sulfinic acid) residues of 2-Cys-Prxs [70] thereby restoring their peroxidase function [32, 71]. Based on studies in yeast, the rate constant for the reduction of oxidized Prx by Trx (about 106 M^−1^ s^−1^) is much faster than the rate of reduction of hyperoxidized Prx by Srx [72, 73]. Thus, the reduction of hyperoxidized Prx by Srx might be considered as a rate limiting step. Moreover Srxs are involved in deglutathionylation processes [74] and can regulate the chaperone function of Prx1 by controlling its glutathionylation levels at position cysteine 83 [75]. In contrast to its anti-oxidant function, which is highly specific for Prxs, the deglutathionylation activity of Srx appears much less restricted [51]. The Srx promoter contains a sequence resembling the consensus sequence for ARE, which is important for its regulation [76]. In response to cigarette smoke and under hypoxic conditions, Srx expression is transcriptionally controlled in a Nrf2-dependent manner [77, 78]. By using overexpression and knock out model systems it has been demonstrated that upon treatment with the chemopreventive Nrf2 inducer 3H-1,2-dithiole-3-thione (D3T) the expression of Srx is upregulated and thus prevents the double oxidation of Prx in neurons [79]. Moreover, hyperoxia has been shown to induce the degradation of mitochondrial double oxidized Prx3 in Nrf2-deficient, but not in WT mice. Thus, in the absence of Srx hyperoxidized Prx becomes susceptible to proteolysis [78]. In addition, the disparate resistance of colon carcinoma cells to ROS has been linked to higher basal levels of Nrf2 and Srx as well as to their distinct cellular localizations [56, 80].

#### Thioredoxin / thioredoxin reductase / TXNIP system

**Fig. 5:**
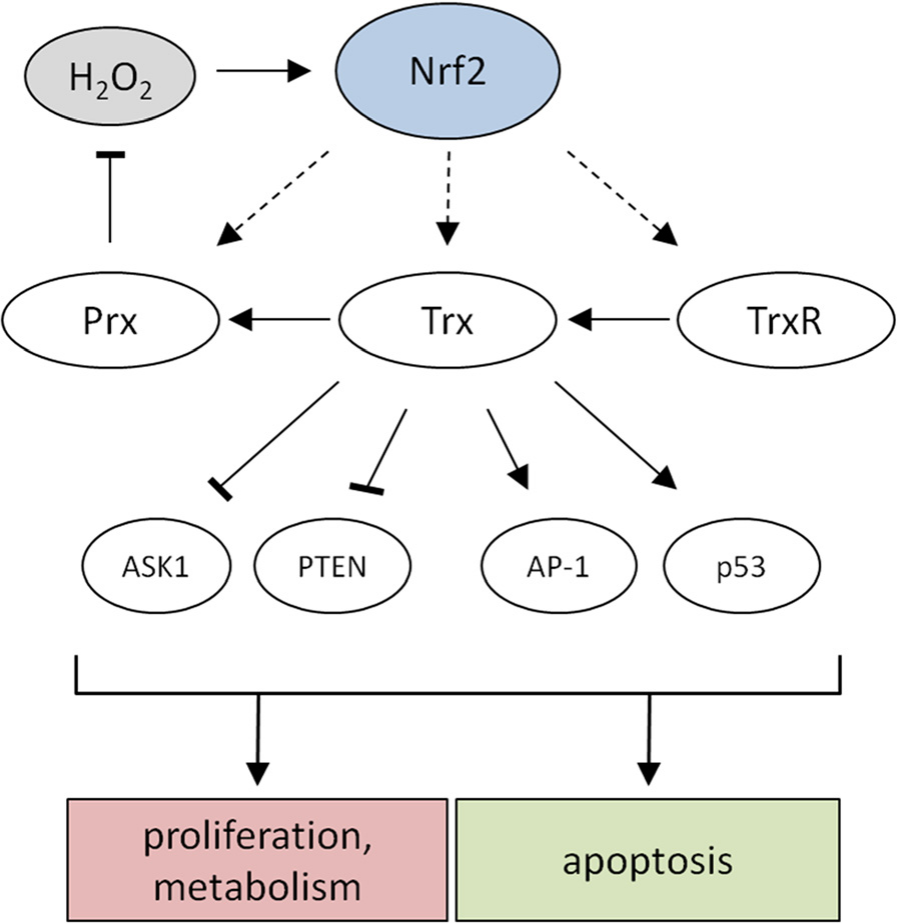
Trx-based upregulation of anti-oxidative systems by Nrf2. Oxidized Trxs are reduced by TrxRs and maintained in their active form. Reduced Trxs can reduce oxidized Prxs, which under physiological conditions detoxify H_2_O_2_. Reduced Trxs can interact with redox-sensitive proteins, such as ASK1, PTEN, AP-1 and p53 suggesting that different cellular processes such as proliferation, the cellular metabolism and apoptosis and might be regulated by Trxs.

Trxs are small ubiquitously expressed proteins maintaining the cellular environment in a reduced state [81]. Trxs are involved in the catalysis of redox-dependent reactions, display oxidoreductase activity, serve as electron donors for enzymes with biosynthetic properties [82] and are involved in the transcriptional control of diverse physiologic and pathophysiologic processes such as cell growth [83], proliferation [84], apoptosis [85] and inflammation [86]. Under physiological conditions Trx is fully reduced and interacts with pro-apoptotic proteins, such as apoptosis signaling kinase 1 (ASK1), the tumor suppressor phosphatase and tensin homologue deleted on chromosome 10 (PTEN), activator protein 1 (AP-1) and p53 [87–89]. In general reversible oxidized redox sensitive cysteine residues of proteins are largely dependent on Trx, which restores their reduced state. However, the enzymatic activity of Trxs relies on the activity of thioredoxin reductases (TrxR), which are selenoproteins and reduce oxidized Trxs under consumption of NAD(P)H [90–93]. Thus, at least baseline activities of TrxR are necessary for cell survival (Fig. 5). As a consequence TrxR might serve as a potential target for cancer treatment by its targeting with electrophilic compounds, which might interact with the redox-active moiety of TrxR [94]. In contrast, the thioredoxin interacting protein (TXNIP) inhibits Trx by binding to its catalytic site thereby competing with other proteins such as ASK1 resulting in an increased susceptibility to undergo apoptosis. In addition, low TXNIP expression correlates with an enhanced tumorigenicity [95] and increased metastasis formation [96].

#### Glutathione system

The glutathione (GSH) system is a major thiol-based defense system against oxidative and electrophilic stress in mammals and functions as co-substrate for the GPxs, which efficiently remove H_2_O_2_ thereby preventing oxidative insults and influencing together with glutaredoxin (Grx) the redox state of proteins via reversible S-glutathionylation [97]. Thus GSH plays an important role in redox-signaling and in the regulation of protein functions. In addition key enzymes of the GSH biosynthesis can be upregulated by Nrf2 [98].

## The specific role of H_2_O_2_ in cancer

**Fig. 6:**
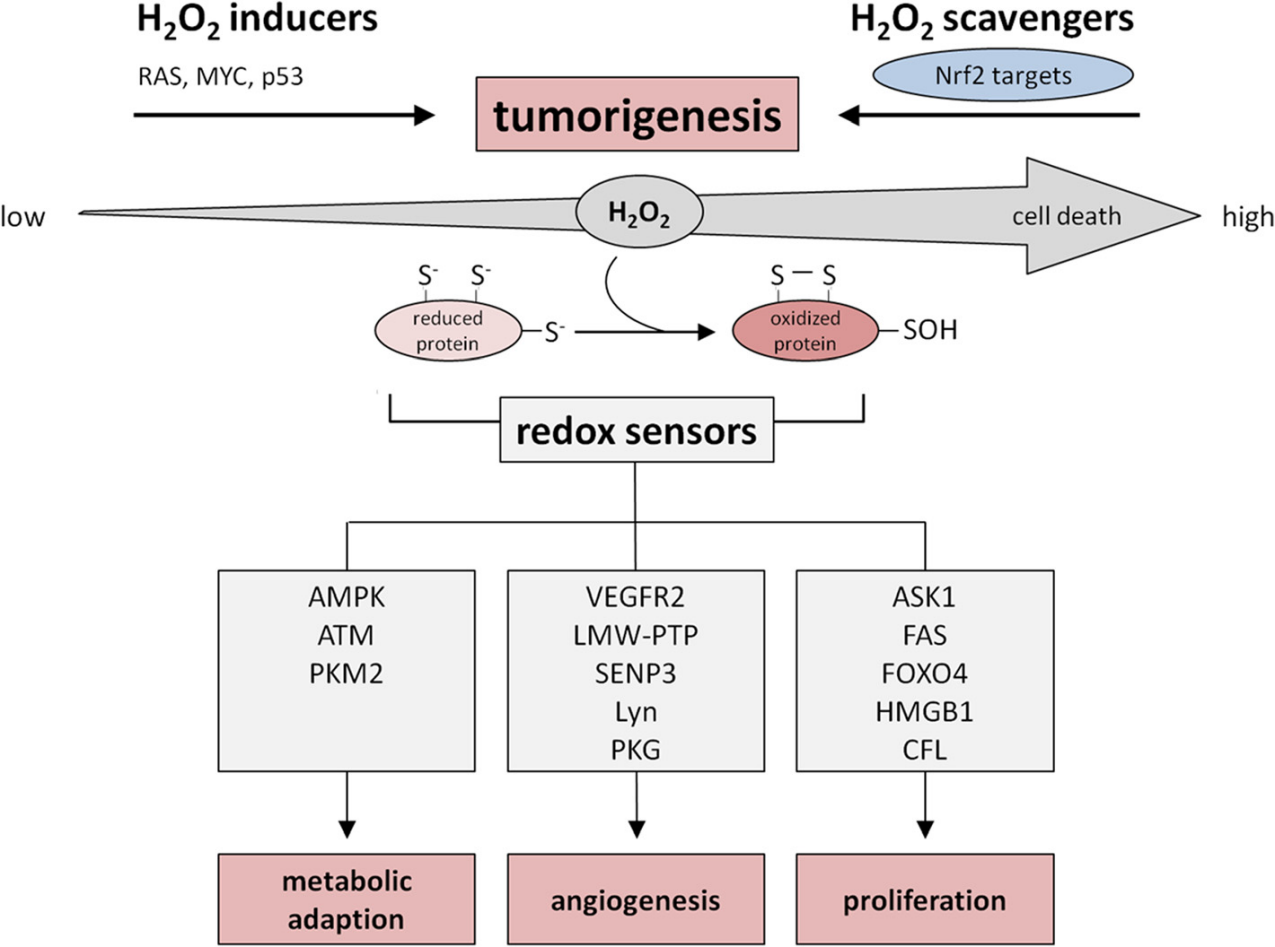
Components of anti-oxidative systems involved in tumor development. Cancer cells are characterized by high levels of ROS (H_2_O_2_). To prevent cell damage and cell death cancer cells induce the expression of anti-oxidative enzymes via the activation of the transcription factor Nrf2. Despite high H_2_O_2_ levels cancer cells maintain the capacity to promote cell survival, differentiation and proliferation by undergoing metabolic adaption processes thereby relying on the redox regulation of cancer-related redox sensors.

Whereas low ROS levels seem to be relevant for the maintenance of cellular homeostasis in normal cells, most cancer cells show metabolic alterations resulting in significantly higher ROS levels, which can trigger either pro- as well as anti-tumorigenic processes. The increased levels of ROS can promote pro-survival and pro-proliferative pathways as well as the metabolic adaption of tumor cells to the tumor environment [99]. The latter includes phosphatidylinositol 3-kinase (PI3K)/AKT/mammalian target of rapamycin (mTOR) resulting at least in part in an increased mitochondrial metabolism [100] along with the inhibition of the anti-oxidative response by phosphorylating members of the fork head box O transcription factor (FOXO) family [101, 102], of the mitogen-activated kinase (MAPK/ERK) as well as of the hypoxia-inducible factor (HIF) signaling cascades [103–105]. Moreover, several oncogenes linked to these pathways, such as RAS, MYC and AKT as well as mutations or loss of tumor suppressors like p53, are associated with increased ROS levels [106, 107]. Yet, increased spatially localized ROS levels can also promote cell toxicity thereby leading to the activation of cell cycle arrest or cell death-inducing pathways resulting in the inhibition of cancer progression [108, 109]. Thus cancer cells do not only have to cope with higher ROS levels [110, 111], but also have to maintain their redox balance, which is frequently accomplished by up-regulating anti-oxidants [112]. In addition, the master regulator of the cellular anti-oxidant response Nrf2 can be activated and stabilized by a number of oncogenes, for example PI3K, K-ras or MYC [47, 113], known to drive signaling cascades that mediate cancer cell proliferation and/or survival. Furthermore, primary tumor cells exert not only higher expression levels of ROS scavengers, including Prxs, SODs and GPxs, but also structural alterations of the Nrf2 inhibitor Keap1 suggesting that an imbalanced redox status promotes tumorigenicity [114–116] (Fig. 6). This is in accordance with an enhanced tumor progression rate in response to treatment with anti-oxidants [117] and an increased resistance to chemotherapeutic drugs via the activation of the Nrf2 [118]. In this context it is noteworthy that several hallmarks of cancer can be directly linked to an increased ROS production [119], such as sustained proliferative signaling [99], resistance to cell death [120], activation of invasion and metastasis [121] as well as induction of angiogenesis [122]. The role of H_2_O_2_ as a promoter of neoplastic transformation is supported by the modulation of the PI3K/AKT signaling pathway due to oxidation of the PTP1B [123, 124] and of PTEN [125] and supported by the inhibition of its induction in the presence of anti-oxidant scavengers [126]. Regarding the tumor cell survival next to the hyperactivation of the PI3K/AKT and K-ras signaling pathways the activation and stabilization of Nrf2 is important for the protection of cancer cells from oxidative stress.

## Correlation of redox-sensitive proteins with neoplastic transformation

H_2_O_2_-mediated signaling events have been reported to affect major features of the cancer cell behavior. Since H_2_O_2_ is involved in the regulation of apoptosis, cell cycle progression and proliferation, the energy metabolism and angiogenesis, specific redox-sensitive targets with redox-sensor functions are necessary (Fig. 6, Table 2).

**Table 2:** Redox-sensitive proteins involved in the regulation of cell metabolism, angiogenesis and cell death

protein	category	modification^a^	cysteine residue	effect
anti-oxidative defense-related redox sensors				
Keap1	others	S-S	C151	inactivation [238]
2-cys Prx	peroxidase	sulfonic acid	C51	inactivation [239]
Trx	others	S-S	C32/C35	inactivation [240]
metabolism-related redox sensors				
AMPK	kinase	G	C299, C304	activation [241]
		S-S	C130, C174	inactivation [132]
ATM	kinase	S-S	C2291	activation [138, 142]
PKM2	kinase		C358	inactivation [146]
angiogenesis-related redox sensors				
VEGFR	receptor	S-S	C1206/C1199	inactivation [147, 148]
LMW-PTP	phosphatase	S-S	C12/C17	inactivation [242]
SENP3	sumo		C243 and/or C274	stabilization [158]
	protease		C243	HIF-1 transactivation [160, 161]
			C532	inactivation, suppressed HIF-1 activity [160, 161]
Lyn	kinase		C466	recruitment of leukocytes [168, 169]
PKG	kinase	S-S	C42	activation [172]
cell death-related redox sensors				
ASK1	kinase	S-S	C250	inactivation [178, 179]
Fas	receptor	G	C294	aggregation, FasL binding [186]
FOXO4	TF	S-S	C477	inactivation [189]
HMGB1	others	S-S	C23/C45	induction of apoptosis [193]
CFL	others	S-S, G	C139/C147, C39/C80	inactivation, inhibition of apoptosis [208–210]

^a^ *C* cysteine, *S-S* intra-/intermolecular disulfide, *G* glutathionylation

### Redox control of the cellular energy metabolism with the relation to cellular growth

**Fig. 7:**
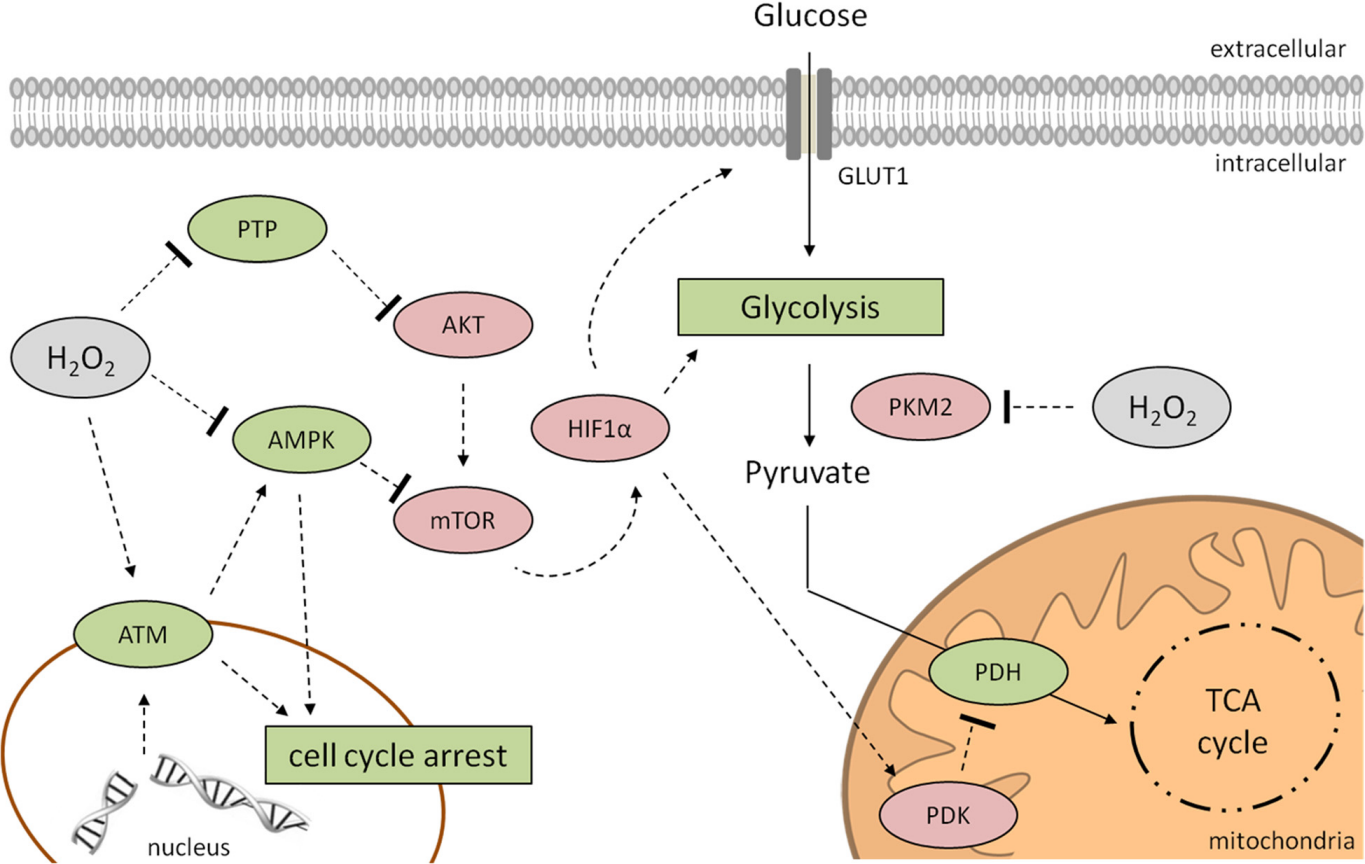
Redox control of the cellular energy metabolism. In cancer cells the shift of the metabolism into anaerobic glycolysis is mainly mediated by the PI3K/AKT pathway. AKT activates mTOR, which subsequently activates HIF1α resulting in an induction of GLUT1, enzymes of the glycolysis and the mitochondrial PDK, which inhibits the pyruvate flux into the TCA. The AMPK is able to block this mechanism by inhibition of mTOR to conserve energy. Cancer cells exhibit high ROS (H_2_O_2_) levels leading to an inhibition of the AMPK and of PTPs, which can inactivate AKT. Even through high H_2_O_2_ levels DSBs could occur leading to the activation of ATM accompanied with cell cycle arrest. The interaction of ATM and AMPK might enhance the DNA damage response. In addition H_2_O_2_ might inactivate the PKM2 leading to an altered flux of glucose in the pentose phosphate pathway for the generation of reductions equivalents to detoxify ROS. PDK, pyruvate dehydrogenase kinase; PKM2, pyruvate kinase M2; TCA, tricarboxylic acid.

In comparison to non-malignant normal cells, cancer cells shift their metabolism to anaerobic glycolysis, which is driven by multiple oncogenic pathways. The PI3K-driven AKT activation leads to a direct regulation of glycolytic enzymes and activation of mTOR. This has an effect on (i) glycolytic enzymes by activation of HIF and/or (ii) induction of the glucose transporter GLUT1, enzymes of the glycolysis as well as the mitochondrial PDK, which inhibits the flux of pyruvate into the TCA [110] (Fig. 7). AMPK can act as an energy sensor protein kinase and opposes this effect by blocking the mTOR activity. Therefore AMPK regulates the energy metabolism by activating energy-producing pathways and inhibiting energy-consuming processes in response to low intracellular ATP levels thereby also linking cellular metabolism to growth control and cell polarity [127]. This was further confirmed by the AMPK inducer 5-aminoimidazole-4-carboxyamide ribonucleoside, which inhibits tumor growth *in vitro* and *in vivo,* suggesting that the AMPK signaling pathway might contribute to the suppression of (tumor) growth by acting as a metabolic checkpoint resulting in cell growth arrest in the G1/S phase in the presence of low intracellular ATP levels [128–130]. Thus, AMPK has a strong impact on the proliferation rate of both non-malignant as well as tumor cells [131].

In addition to oncogenic mutations and signaling pathways [128] the AMPK activity can be suppressed by oxidation of cysteine residues within the catalytic subunit alpha at positions 130 and 174 promoting its aggregation. In contrast, the reduction of these sites is required for the successful activation of the AMPK complex during energy starvation, which is mediated by Trx thereby providing evidence that oxidative stress and metabolism can be linked via AMPK [132]. Furthermore, AMPK can function as a sensor of genomic stress and interacts/enhances the DNA damage response by interaction with the serine/threonine protein kinase ATM [133] a redox sensor for the regulation of DNA repair processes. Under physiologic conditions ATM is recruited and activated by DNA double-strand breaks (DSBs) via the formation of MRE11-Rad50-Nibrin (MRN) DNA repair complexes. This results in the phosphorylation of various key proteins involved in DNA repair processes, such as p53, the serine/threonine-protein kinase Chk2 (CHK2) and the histone H2AX (H2AX) [134–137]. In the presence of H_2_O_2_ ATM forms a disulfide-cross-linked dimer resulting in its direct activation independent from the MRN complex formation thereby supporting its redox sensor function [138]. Furthermore, ATM is involved in the regulation of mitochondrial function and metabolic control by interaction with p53, AMPK, mTOR and HIF1α [139–141], which is independent of DSBs [142]. In addition, the redox status of tumors functions as a major determinant of the ATM-dependent molecular switch of resistance to apoptosis. At low ROS levels apoptosis was blocked, whereas increased cellular ROS levels restored ATM/JNK-mediated apoptotic signaling [143]. There is also evidence that pathological neoangiogenesis requires ATM-mediated oxidative defense, since agents promoting excessive ROS generation have beneficial effects in the treatment of neovascular diseases [144]. Not only AMPK, but also the pyruvate kinase isoform M2 (PKM2), known to be over-expressed in tumors [110], represents a switch between glycolysis and gluconeogenesis. Inhibition of PKM2 caused by oxidative modification of the cysteine residue at position 358 [145] contributes to maintain cellular anti-oxidant responses by diverting the glucose flux into the pentose phosphate pathway thereby generating sufficient reducing potential for the detoxification of ROS [146].

### Redox control of cellular signaling processes in association with angiogenesis and cell death

ROS, which are generated in response to various stimuli including growth factors, have been shown to modulate cellular growth and angiogenesis. A major source for ROS are NOX enzymes that can be activated by various growth factors, e.g. vascular endothelial growth factor (VEGF) and angiopoietin-1, leading to the induction of genes involved in angiogenesis and thus represent therapeutic targets for the inhibition of tumor angiogenesis [122]. H_2_O_2_ derived from NOX activities can affect the vascular endothelial growth factor receptor (VEGFR) 2, which regulates angiogenesis, vascular development, vascular permeability and embryonic hematopoiesis, but also promotes cell proliferation, survival, migration, and differentiation of vascular endothelial cells. Despite VEGFR1 and VEGFR2 can bind VEGFA, VEGFR2 plays the major role in modulating these processes. Its activation depends not only on the autophosphorylation of defined tyrosine residues, but is also regulated by oxidative modifications [147, 148]. Increased cellular H_2_O_2_ levels promote the formation of an intracellular disulfide bond thereby blocking the receptor activity, whereas the presence of Prx2 effectively prevents this oxidative modification leaving the receptor responsive to VEGFA stimulation [147, 148]. Furthermore, extracellular H_2_O_2_ generated by extracellular SOD promotes VEGFR2 signaling via oxidative inactivation of protein tyrosine phosphatases (PTPs) in mice [149]. Moreover, the expression of TXNIP is required for the VEGF-mediated VEGFR2 activation and angiogenic response *in vivo* and *in vitro* by regulating VEGFR2 phosphorylation via *S*-glutathionylation of the low molecular weight protein tyrosine phosphatase (LMW-PTP) in endothelial cells [150]. In addition the interaction of TXNIP with the poly-ADP-ribose polymerase 1 (PARP1) is a relevant regulator for its translocalization and function leading to the activation of VEFGR2 signaling in human umbilical vein endothelial cells [151]. Furthermore, H_2_O_2_ was shown to induce the expression levels of the VEGFR2 ligand VEGF by inducing the transcription factors NFκB or AP-1 [152]. Under hypoxic conditions VEGF expression is upregulated by HIF1α which is over-expressed in many tumors and its activity levels influence angiogenesis as well as tumorigenesis [153]. Under normoxic conditions HIF1α is hydroxylated and subsequently ubiquitinated for proteasomal degradation, whereas under hypoxic conditions its hydroxylation is blocked leading to its accumulation, dimerization with its beta subunit and subsequent translocation into the nucleus, where it regulates the expression of genes linked to cellular transformation, cell proliferation and angiogenesis [154–156]. The transcriptional activity of HIF1α depends on the translocation of sentrin/SUMO-specific protease 3 (SENP3) from the nucleoli to the nucleoplasm [157]. ROS seem to be involved in limiting its proteasomal degradation. The complex formation with either the heat shock protein 90 (Hsp90) or the co-chaperone/ubiquitin ligase carboxyl terminus of Hsc70-interacting protein (CHIP) leads to the stabilization or degradation of SENP3. Under mild oxidative stress the oxidation of thiol residues favors the recruitment of Hsp90 thereby protecting SENP3 from binding to CHIP, which results in its ubiquitination and subsequent elimination via proteasomal degradation. Thus, the redox status of SENP3 is a decisive factor for its stabilization or degradation [158] and can regulate the expression of the EMT-inducing transcription factor fork head box C2 (FOXC2) that is de-SUMOylated and thereby activated in response to increased ROS levels. As a result the expression of the mesenchymal marker protein N-cadherin is induced [159]. In HeLa cells ROS levels are involved in the activation of HIF1α by modifying cysteine residues at positions 243 and 532 of SENP3 thereby controlling the interaction of SENP3 with p300, the co-activator of HIF1α. This is accompanied by SUMOylation of p300 resulting in the transcriptional silencing of HIF1α. The shift of HIF1α transactivation by ROS depends on the biphasic redox sensing of SENP3. Whereas low ROS levels lead to SENP3 accumulation and therefore enhanced HIF1α transcriptional activity, high concentrations of ROS inactivated SENP3 resulting in the suppression of HIF1α transcriptional activity. Thus SENP3 is an example for a redox sensitive protein with cysteine residues that can sense different ROS levels [160, 161]. VEGF can also promote endothelial permeability through the activation of the Src family non-receptor tyrosine kinases (SFKs) [162]. Lyn, a member of the SFK family, has been shown to be amplified and upregulated in tumor cells, which is associated with resistance to chemotherapy [163] and plays an important role in the regulation of both innate and adaptive anti-tumoral immune responses. Since NOX-expressing tumors are able to efficiently produce H_2_O_2_, the tumor stroma can mimic features of ‘unhealed’ wounds [164]. Using distinct model systems, extracellular H_2_O_2_ levels have been linked to the recruitment of leukocytes, such as neutrophils, representing the first line of innate immune responses [165–167]. In addition, Lyn serves as a redox sensor for neutrophils monitoring the redox state of wounds. The oxidation-specific modification site was defined as cysteine residue 466, which directly triggered the wound response and calcium signaling [168, 169]. In response to treatment with chromium (V) complexes the formation of ROS and activation of Lyn were found in lymphocytes leading to the activation of caspase-3 and subsequently to the induction of apoptosis [170]. Another kinase with redox-sensor function and involvement in angiogenesis is the cGMP-dependent protein kinase (PKG). PKG represents a member of a serine/threonine-specific protein kinase family that acts as a key mediator of the nitric oxide (NO)/cGMP signaling pathway. GMP binding has been shown to activate PKG resulting in the phosphorylation of serine and threonine residues on many cellular proteins [171] involved in modulating cellular calcium. Besides this activation mechanism it is also known that PKG can be activated under oxidative stress independent of the respective cGMP or NO levels [172]. PKG controls the regulation of platelet activation and adhesion, smooth muscle contraction, cardiac function, gene expression and the feedback of the NO-signaling pathway amongst others. While the expression of PKG in metastatic colon carcinoma blocks tumor angiogenesis by down-regulating the expression level of beta-catenin [173], PKG signaling can also mediate cytoprotective and anti-apoptotic function in various tissues including non-small-cell lung carcinoma. Thus, PKG inhibitors might be of therapeutic relevance and have been suggest for treatment in combination with cisplatin chemotherapy of solid tumors [174]. PKG-inhibitors limit the migration and invasion capacity of colorectal carcinoma cells [175]. Moreover, pro-apoptotic effects of PKG signaling have been reported for various colon carcinoma as well as breast cancer cell lines, which is in line with the hypothesis that the loss of PKG expression in colon carcinoma cell lines may contribute their resistance to undergo anoikis [176, 177].

### Redox control of cellular signaling processes in association with apoptosis

By acting as a mitogen-activated protein (MAP) kinase kinase kinase (MAPKKK) ASK1 can activate two distinct sets of MAPKK. Whereas the tumor necrosis factor alpha (TNF-α)-mediated activation of MKK4 (SEK1) via its downstream target JNK leads to the induction of apoptotic cell death, the activation of MKK6 activates p38 subgroups of MAPK, which phosphorylate a wide range of potential targets in response to inflammatory cytokines and cellular stress. A key role in the ASK1-mediated induction of apoptosis via MKK is its dimer formation, known to be induced by exposure to H_2_O_2_, but blocked by Trx supporting its role as a redox sensor. Moreover, the interaction of ASK1 and Trx is based on the formation of a disulfide bond at the N-terminal domain of ASK1 leading to its ubiquitination and subsequent proteasomal degradation. However, high levels of H_2_O_2_ caused a loss of the protective function of Trx due to the formation of an intramolecular disulfide bond resulting in its release from ASK1, which is accompanied by its activation [178, 179]. Furthermore, the selective inhibition of TrxR by the drug MC3 or by electrophilic pollutants leads to the induction of apoptosis via the Trx-ASK1-p39 signal cascade by blocking the interaction of Trx with ASK1 [180, 181]. In addition, redox alterations induced by selective inhibition of the glucose metabolism leading to massive oxidative stress might serve as a molecular switch that activates the ASK1-JNK/p38 MAPK signaling pathways accompanied by promotion of the radiosensitization of malignant cells [182]. Similar effects have been reported in response to treatment with iron chelators, which also resulted in reduced ASK1-Trx complex formation [183]. The genetic inhibition of ASK1 resulted not only in the inhibition of JNK activation, but also in decreased expression of Fas ligand (FasL) and subsequent apoptosis, whereas the inhibition of p38 did not alter the FasL expression [184]. The activation of Fas upon ligand engagement leads to the formation of a death-inducing signaling complex accompanied by caspase 8-mediated apoptosis [185]. The Fas/FasL interaction results in the *S*-glutathionylation of Fas at cysteine residue 294 [186], which not only increases the binding to its ligand, but also its aggregation and recruitment into lipid rafts. This oxidative modification can be linked to the activity of Grx1 [187], since the depletion of Grx1 results in an increased *S*-glutathionylation rate along with the induction of apoptosis, while Grx1 overexpression causes opposite effects. The level of oxidative stress mediated by exogenous sources or endogenously generated upon receptor stimulation regulates the sensitivity to Fas-mediated apoptosis [188]. Additionally FOXO4, a TF involved in the regulation of the insulin signaling pathway, can be activated by oxidative stress due to the formation of an intermolecular disulfide bond between cysteine residue 477 and histone acetyltransferase p300 resulting in the formation of a covalently linked heterodimer. The redox modification of FOXO4 is essential for its subsequent CREB-binding protein (CBP)-mediated acetylation [189]. However, the activity of the heterodimeric complex is regulated by the Trx system, which has a strong impact on the turnover of this interaction by reducing the cysteine-dependent heterodimer of FOXO4 and p300 thereby providing evidence that Trx might be a key regulator of ROS-dependent FOXO4 signaling [189]. In addition, the efficient nuclear translocation and subsequent activation of FOXO4 in response to ROS depends on disulfide formation with the nuclear import receptor transportin-1 (TNPO1), whereas its insulin signaling-dependent nuclear shuttling is not dependent on TNPO1 [190]. Although high-mobility group box 1 protein (HMGB1) might act as a redox-sensitive switch between autophagy and apoptosis. HMGB1 is a DNA-binding protein that associates with chromatin, but can also bind single stranded DNA linking the assembly of transcriptional active protein complexes on specific targets. Its reduced form interacts with the receptor for advanced glycation end products (RAGE) thereby inducing beclin1-dependent autophagy [191]. In the presence of higher ROS levels HMGB1 can undergo oxidative modification leading to the formation of a disulfide bond between cysteine residues 23 and 45 [192], which induces apoptosis via the intrinsic pathway [193]. When released in its partially oxidized status, HMGB1 functions as a pro-inflammatory cytokine [194], whereas in its fully oxidized form (sulfonylated) all biologic activities are lost. Furthermore, HMGB1 can be released from both activated and dying cells thereby acting as a damage-associated molecular pattern molecule [195]. However, its biochemical and immunological properties depend on both its cellular localization as well as its release mechanism [196]. Due to different intracellular and extracellular functions HMGB1 is a central mediator in inflammation and immunity, but its activity depends on the state of its redox-sensitive cysteine residues at positions 23, 45 and 106 ranging from DNA binding, to induction of chemotaxis and transcription of chemokines [197, 198] suggesting its classification as an “alarmin” for sepsis and cancer [199]. Different diseases, such as cancer, are often accompanied by T cell hyporesponsiveness, which is mediated by ROS. The release of H_2_O_2_ produced by tumor-infiltrating macrophages leads to the suppression of potentially tumor reactive T cells [200]. Cofilin (CFL), a member of the actin-depolymerizing factor protein family, binds to F-actin and plays an important role in the regulation of the actin cytoskeleton dynamics as well as in the mitochondrial apoptosis. Its translocation from the cytoplasm into the mitochondria leads to cytochrome c release and activation of caspase signaling, thus representing an early step in the induction of apoptosis [201, 202]. Since CFL is also associated with invasion and metastatic capacity of tumors [203–206], it is a key therapeutic target for tumors [207]. CFL might function as a redox sensor [208] and its dephosphorylation-dependent glutathionylation [209, 210] not only leads to a loss of its actin binding affinity, but also blocks its translocation to the mitochondria thereby preventing apoptosis induction. The oxidation-mediated inactivation of CFL can also provoke T cell hyporesponsiveness or the necrotic-like programmed cell death, which modulates the T cell activation processes including the duration of the effectors phase [211]. In contrast, knockdown of CFL could protect T cells from fatal effects of long-term oxidative stress [212] suggesting that oxidation and mitochondrial localization of CFL represents a check point for necrotic-like cell death. Therefore the oxidation of CFL might provide a molecular explanation for the T cell hyporesponsiveness found in diseases such as cancer under oxidative stress conditions [200].

## Components of redox regulating processes as therapeutic targets

Tumor cells take the advantage of upregulating anti-oxidant systems to protect themselves against ROS-induced cell damage. The upregulation of anti-oxidant molecules is often associated with an increased cell proliferation, survival and chemotherapy resistance. Therefore components of the anti-oxidant processes including the Trx system represent potential therapeutic targets for the treatment of cancer patients to trigger ROS mediated cell death (Table 3). This is in line with the reduced tumor cell proliferation, induced apoptosis and increased sensitivity of tumor cells to anti-cancer therapy in the presence of Trx and TrxR1 inhibitors [213, 214]. Since a cross-talk between different anti-oxidant molecules has been shown, a combinatorial targeting of these molecules is essential for complete inhibition of the anti-oxidant defense system. Indeed inhibition of TrxR in combination with the disruption of the GSH biosynthesis, caused a selective cell death of human head, neck, and lung cancer cells by inducing oxidative stress [215, 216]. In addition to targeting the Trx system other molecules, e.g. the inhibition of the PTEN axis [217] or the protein deglycase DJ-1 (DJ-1) known to exhibit anti-oxidative and cyto-protective functions in other diseases [218–220] might have therapeutic potential, since DJ-1 is often upregulated in cancer cells and involved in the regulation of various redox stress responsive signaling pathways (PI3K/AKT/PKB; Trx1/ASK1) [217, 221]. Thus, a combined targeting of Trx and DJ-1 result in a complete loss of the anti-oxidant defense system [217].

**Table 3:** Current therapeutic strategies for the treatment of cancer patients to trigger ROS-mediated cell death

substance	target/mechanism	treatment of
Auranofin	TrxR inhibitor	leukemia, solid cancer, melanoma [213]
+ BSO	inhibition of GSH biosynthesis	head and neck cancer cells, malignant B cells [215, 216]
+ Selenocystin		lung cancer [214]
MJ25	TrxR inhibitor	melanoma [213]
Imexon	prooxidant, cysteine depletion	B cell non-Hodgkin lymphoma [243, 244]
Calmangafodipin	SOD mimetica	metastatic colorectal cancer [245]
Molexafin gadolinum	TrxR inhibitor, superoxide formation	glioblastoma multiform [246]
Arsenic trioxide	inhibitor of mitochondrial chain, GPx and TrxR	melanoma [247]
+ GSH depletion		

However, the interaction between different anti-oxidant molecules in distinct tumor models requests further analysis to increase the insights of the underlying molecular mechanisms of these interactions and the identification of additional molecular targets for cancer therapy. In addition, a better understanding of the role of the intracellular redox state balance and the redox-regulated signaling cascades might enhance the therapeutic options for the treatment of various human cancer types.

## Conclusions

Many cancer cells are characterized by an increased intrinsic formation of ROS as a result of their malignant transformation process. Yet, they have to adapt to this challenge in order to maintain the capacity for tumor progression. ROS, in particular H_2_O_2_, play an important role in facilitating both cell proliferation and cell survival of tumor cells by triggering the redox signaling cascades. New therapeutic approaches are currently developed that aim towards altering the tumor cell redox state, including (i) the selective inhibition of cellular ROS sources [222, 223], e.g. NOX, (ii) the hyperactivation of anti-oxidant enzymes to lower intracellular ROS levels and (iii) the modulation of the anti-oxidant response system towards increasing ROS levels thereby further promoting the induction of apoptosis. So far, the underlying molecular mechanisms of the interactions between different redox signaling compounds and the tumor progression processes are not fully understood. In addition, there is still a need to define additional redox sensors. Therefore, further research is required to gain additional insights into these signaling networks and sensors, which then might lead to the identification and subsequent design of novel targeted therapies for the treatment of cancer patients.

## Abbreviations

AMPK: AMP-activated protein kinase
AKT: Protein kinase B
AQP: Aquaporin
ARE: Anti-oxidant response element
ASK1: Apoptosis signal-regulating kinase 1
ATM: Ataxia telangiectasia mutated
CFL: Cofilin
EMT: Epithelial-mesenchymal transition
ERK: Extracellular signal regulated kinase
Fas: Tumor necrosis factor receptor superfamily member 6
GPx: Glutathione peroxidase
Grx: Glutaredoxin
GSH: Glutathione
GST: Glutathione S transferase
H_2_O_2_: Hydrogen peroxide
HIF: Hypoxia inducible factor
HMGB1: High-mobility group box 1 protein
JNK: c-Jun amino-terminal kinase
Keap1: Kelch-like ECH-associated protein 1
MAPK: Mitogen-activated protein kinase
mTOR: Mammalian target of rapamycin
NO: Nitric oxide
NOX: NAD(P)H oxidase
NFκB: Nuclear factor-kappaB
Nrf2: Nuclear factor-erythroid 2 p45-related factor 2
O_2_^−^: Superoxide anion
PI3K: Phosphatidylinositol 3-kinase
PKG: cGMP-dependent protein kinase
PKM2: Pyruvate kinase M2
Prx: Peroxiredoxin
PTEN: Phosphatase and tensin homologue deleted on chromosome 10
PTP: Protein tyrosine phosphatase
ROS: Reactive oxygen species
SENP3: Sentrin/SUMO-specific protease 3
SOD: Superoxide dismutase
Srx: Sulfiredoxin
TF: Transcription factor
TNF-α: Tumor necrosis factor alpha
Trx: Thioredoxin
TrxR: Thioredoxin reductase
TXNIP: Thioredoxin interacting protein
VEGF: Vascular endothelial growth factor
VEGFR2: Vascular endothelial growth factor receptor 2
